# Cancrum or Scirrhus Gangrenousa of the Inferior Maxillary

**Published:** 1888-08

**Authors:** J. T. McMillan

**Affiliations:** Paris, Ky.


### THE DENTAL REGISTER.

Vol. XLII.] AUGUST, 1888. [No. 8.

### Communications.

### Cancrum or Scirrhus Gangrenousa of the Inferior Maxillary.

BY J. T. MCMILLAN, OF PARIS, KY.

In attempting to give as complete description of this case, as should be done—having been associated with three physicians, it (I fear) would necessitate so many minute details, that it would fail to interest those that have not seen similar troubles. The case becomes the more interesting the more it is studied. We have called this trouble Cancrum, or Scirrhus, because of the dried up or parched condition in which we find the edges of the diseased growth. We cannot say that it is cancer, although malignant in appearance; nor could we say what the termination would have been without the presence of gangrene. Take all the authors who have written on gangrenous or cancerous conditions of the oral cavity, and see the fatality in the great number of cases. Take the experience of the best practitioners, who have had the advantage of hospital practice and see what they say. There are not more than five in every one hundred cases of adults who suffer with gangrene of the mouth that are cured, and these in most cases are left with unsightly deformities.

Miss S., aged 23 years; bilio nervous temperament, persistent nerve force and marked determination, called at my office October 14th, 1886, complaining of some pain in her face in region of ear. It had been my pleasure to have the care of her teeth since a child. I had seen developed an unusually well formed set of permanent teeth, and to this age she had only a few small crown fillings.

The examination showed no signs of disease, but barely a slight sensitiveness from percussion of an instrument in the second inferior right molar. She had felt this pain at intervals for a week past. A diagnosis not giving sufficient evidence of trouble in the dental organs, advised her to call on her physician for systemic treatment.

Patient called again on the 16th insisting that there was something the matter with her teeth, and she apprehended something of a very serious nature, that she could not sleep, and was in constant pain. It was something so unusual to hear *her* complain. We made a very careful and complete examination; found the second molar as before slightly sensitive to the touch, but firm. The gums and the soft parts on the buccal surface from where the wisdom tooth should erupt forward to the second bicuspid presented an appearance as if it had been stippled with an instrument but looked hard and slightly swollen and when touched exuded a glutinous sticky discharge. Not being thoroughly satisfied mopped the parts with a solution of carbolic acid and advised to see her physician. She went home but suffered so much from pain called physician during the night. The physician, Dr. H. call me on the 18th. Examination, pulse 120. Patient complained of severe and acute pain in side of face, neck and shoulder. Not able to locate it at any definite point; face seemed swollen on the out side; the interior of the mouth on the buccal surface, running from the first bicuspid to, and back of the second molar presented a great number of pustules, resembling in appearance and shape frogstools or something of a mushroom growth, of a steel-grayish color. These pustules seem to have come from what appeared to be the stippled places on the gum; while soft, were dry and crusty on their edges, of a tenaceous character and hard to remove; after having been removed would recur again in from four to six hours, and looked the same as if they had not been touched.

Patient continued to suffer from pain, and a growing (as she described it) feeling in the right side of the face. October 19th, pulse 130; had passed a night without sleep; constant pain all over the right side of face and head; slight external swelling; the inside of the mouth presented a somewhat irritated condition; tongue swollen and much furred; the same pustules in appearance, but larger, and when scraped off returned much quicker but did not cover as much surface; the largest collection being around the first and second molars; the second molar appeared a little sore when pressed from within outwards.

October 20th. High fever; pulse 132; increased pain extending from inferior maxillary all over the head, neck, shoulders and breast; patient not able to eat or sleep.

October 21st. Drs. E. and B. called in consultation; pulse 136; patient had suffered excruciating pain for the last twelve hours; face swelling very rapidly; tongue slightly swollen; right eye nearly closed and very red; swelling extending from the temporal ridge downward to, and below the collar bone; saliva copious and offensive; severe chill; an indication of formation of pus, or disintegration, suppuration, and destruction of tissues.

October 22d. Pulse 140. Patient very much broken down; had taken no nourishment for four days except stimulants; the swelling had become very hard, and from the outside presented a glossy pale pink color surrounding a deep-seated tumefaction in the cheek. This tumefied portion seemed of round or button shape and directly opposite the point where the frogstool pustules first made their appearance. On the inner surface was an eschared black or grayish-colored spot and discharging a steel-greyish fluid of an almost unbearable fetid odor. This odor or gangrene felon, so permeated the house that it became nauseous and almost unbearable to the inmates. The swelling had progressed so rapidly that the neck was near the size of the head, and it was with great difficulty you could see in the mouth.

Thus within six days we see irritation, disturbance of circulation, stasis, exudation, swelling, chronic inflammation, suppuration, and destruction of tissues.

And what was the irritant?

Patient had retained a normal mental condition until this time.

During these six days of suffering the pain had been alleviated to some extent by the use of morphia taken internally and hypodermically with stimulants every hour, and increased in quantity until the tenth day when there was as much as a quart of whisky taken daily—and this great quantity never in the least impaired the mental condition.

TREATMENT.

On October 23d or the 7th day. (The soft parts of the inferior maxillary, and also of the inner surface of the cheek, the mucous membrane not being broken except by the pustules and the discharge from them). We made an incision from where the wisdom tooth should erupt forward to the mental foramen and perpendicularly to, and below the mental foramen. This incision revealed a great quantity of disintegrated tissues of a dark grayish color, together with an occasional gush of clear gelatinous serum. This virus wherever it touched healthy tissue, was death to it. The pus had burrowed into pockets as far down as the line of the facial artery and posteriorly to the external carotid artery. The external plate of the alveolar process, around the second bicuspid, first and second molars was *necrosed.* The necrosis extended deep into the maxillary, around the second, and between the first and second molars, necessitating the extraction of the second molar, which was partially necrosed to within three or four lines of the apex of root. Cut away all the necrosed bone with a plate scraper and hoe-shaped excavator. After cleansing as well as possible applied sulphuric acid from where the necrosed bone had been taken by means of a drop or safety syringe. After protecting the healthy parts of the mouth applied solution of chromic acid and syringed out with solution of permanganate of potassium. Not having the desired effect cauterized the affected parts thoroughly with nitric acid. During this operation there was no indication of hemorrhage except a small quantity from the lingual surface when the tooth was extracted. Patient experienced but little pain from the operation. The application of nitric acid was kept up once every twenty-four hours for three days—four times in twenty-four hours. The pockets and dead surfaces were dressed with glycerine and iodine. Patient had retained strength and seemed at times bright until the eleventh day; the stomach refused stimulants and nausea set in. The systemic treatment principally was iron and quinia in connection with whisky. After the fifteenth day healthy granulation was perceptable, and at times when the exposed surfaces were touched there were blood traces visible—that ever present fetid odor which had been kept down, to some extent by use of disinfectants, now commenced to subside. The scurf from the charred and crisped action of the cautery commenced to loosen making the parts very sore to the touch. On the seventeeth day the escharred button came loose from the cheek and revealed a line of demarcation between healthy and diseased tissues. The eighteenth day this button crust was removed and when the bulb of a burnisher was introduced on the inside from where this escharred crust came, and your finger placed on the cheek on the outside, it felt as no more than the thickness of paper between them. The pockets which the burrowing of the pus had made, were well syringed three times daily with a strong solution of carbolic acid alternated with tincture of calendula. We saw the patient twice a day for this purpose until December 12 when the pockets were sufficiently closed not to need constant attention.

Patient was discharged December 28th with no defect except a slight depression in the cheek, which is fast filling up.

